# Epidemiology and Clinical Spectrum of Human Bocavirus Infection in Children: A Retrospective Single Center Analysis

**DOI:** 10.3390/v18101071

**Published:** 2026-09-28

**Authors:** Sanda Škrbina, Dominik Ljubas, Ivana Valenčak, Ivana Ferenčak, Irena Tabain, Srđan Roglić, Goran Tešović

**Affiliations:** 1 Pediatric Infectious Disease Department, University Hospital for Infectious Diseases ‘Dr. Fran Mihaljević’, Mirogojska 8, 10000 Zagreb, Croatia; sskrbina@bfm.hr (S.Š.); dljubas@bfm.hr (D.L.); ivalencak@bfm.hr (I.V.); sroglic@bfm.hr (S.R.); 2 Department of Microbiology, Croatian Institute of Public Health, Rockefeller Street 7, 10000 Zagreb, Croatia; ivana.ferencak@hzjz.hr (I.F.); irena.tabain@hzjz.hr (I.T.); 3 School of Medicine, University of Zagreb, Šalata 3, 10000 Zagreb, Croatia

**Keywords:** human bocavirus, pediatric, coinfection, respiratory tract illness, pneumonia

## Abstract

**Background:** Human bocavirus is a non-enveloped DNA virus of the *Parvoviridae* family, first identified in 2005 and is recognized as a respiratory pathogen across pediatric age groups, with the highest prevalence reported in children aged 6–24 months. Interpretation of HBoV infection is complicated by frequent viral co-detection and the lack of a reliable method to distinguish active infection from prolonged viral sheding. **Methods:** We analyzed epidemiological trends and viral co-detection rates, along with demographic, clinical, laboratory, and radiological features of patients aged 0–18 years infected with HBoV during a four-year period (2022–2025) at a tertiary pediatric center in Croatia. **Results:** Of 27,361 respiratory samples collected, 1143 (4.2%) were positive for HBoV. Annual detection rates remained stable (3.6–4.8%), with predominant winter circulation. Viral co-detection was frequent (71.0–82.7%), with adenovirus and rhinovirus being the most commonly co-detected viruses. Pneumonia was diagnosed in 23.7% of patients, with age > 24 months (aOR 2.32; *p* = 0.012) and higher leukocyte count (aOR 1.13; *p* < 0.001) recognized as associated factors. Severe disease was uncommon: seven patients required oxygen support, and four were admitted to the PICU. Around one-quarter had gastrointestinal manifestations. Prolonged HBoV detection was observed, with the longest interval being 291 days. **Conclusions:** This is the largest single-center study of HBoV infection in the general pediatric population in Croatia. HBoV was predominantly detected in toddlers, with frequent viral co-detection, although HBoV-related pneumonia was more common in older children. Further studies are needed to clarify the role of HBoV in respiratory illness.

## 1. Introduction

Human bocavirus (HBoV) is a non-enveloped, single-stranded DNA virus belonging to the *Parvoviridae* family, *Parvovirinae* subfamily, and the genus *Bocaparvovirus*, which comprises viruses that infect only vertebrates. It was first discovered in 2005 from the samples of nasopharyngeal aspirates, collected from children with upper (URTI) and lower respiratory tract infection (LRTI) [1,2]. The first identified serotype, HBoV1 has been recognized as a potential respiratory pathogen, whereas the additionally identified serotypes 2–4 were detected in stool samples and are associated with acute gastroenterocolitis [3]. Transmission occurs primarily through the respiratory route, as well as through direct ingestion [4]. HBoV has been detected across all age groups, but it most commonly affects children aged 6 to 24 months and the immunocompromised [3]. It typically presents as respiratory illness, including the common cold, acute otitis media (AOM), bronchiolitis, bronchitis, asthma exacerbations, pneumonia, and acute gastroenteritis [4,5,6]. Although HBoV infection is usually mild and self-limiting, rare and potentially life-threatening lower respiratory tract complications have occasionally been reported, including respiratory failure, pneumomediastinum, pneumothorax and plastic bronchitis [6,7]. In addition, neurologic and cardiac manifestations have been described, including encephalitis and myocarditis, although evidence for these associations remains limited [8,9].

HBoV infections occur throughout the year, with peak incidence varying between geographic regions, but are generally most common during the winter and spring months [2]. In Croatia, similar trends are observed [10]. The diagnosis of the infection is primarily based on nucleic acid detection, using polymerase chain reaction (PCR), as viral isolation is technically demanding and is not routinely performed [2,11]. Documented prolonged viral shedding, a notable proportion of asymptomatic individuals, and considerable proportion of coinfections with other respiratory pathogens complicate the attribution of HBoV as the definitive cause of disease and have led to suggestions that the virus may, in some cases, represent a colonizer, rather than a true pathogen [1,2,12,13]. To date, only few studies among hospitalized pediatric patients have investigated the epidemiology, clinical, and molecular characteristics of HBoV infection in Croatia, pointing out variable prevalence and frequent viral co-detections [10,11,14,15]. Phylogenetic analysis identified HBoV1 as the predominant genotype, while a subsequent study demonstrated an association between higher HBoV viral load and disease severity [10,11].

In this study, we report HBoV epidemiological trends, prevalence and viral co-detection rates among pediatric patients aged 0–18 years, treated at University Hospital for Infectious Diseases (UHID) in Zagreb County during a four-year period (2022–2025) following the introduction of PCR multiplex method into clinical practice. Additionally, we provide an analysis of demographic, clinical, laboratory, and radiological features of patients with HBoV monodetection to explore the role and clinical spectrum of HBoV as a causative agent of respiratory tract infection (RTI) in children.

## 2. Materials and Methods

### 2.1. Patients and Samples

We analyzed HBoV-positive cases and co-detection rates among pediatric patients aged < 18 years old from whom respiratory tract specimens were collected at UHID and analyzed at Croatian Institute of Public Health (CIPH). In cases of repeated HBoV positivity in the same patient during subsequent Emergency Department (ED) visits within the same season, only the first positive specimen was included, under the assumption that repeated positivity represented prolonged viral shedding rather than reinfection. Annual proportions of HBoV-positive cases, along with viral co-detection rates and demographic data were reported for a four-year period, beginning on 1 January 2022 to 31 December 2025. For comparative analyses, this period was further divided into the late pandemic period (January 2022–May 2023) and the early post-pandemic period (June 2023–December 2025), based on the WHO declaration of the end of the COVID-19 public health emergency in May 2023. Based on age, patients were classified into age groups (0–6 months, 7–12 months, 12–24 months, 2–5 years, >5 years old).

We reviewed medical records of patients among whom HBoV was the only detected pathogen from PCR respiratory kit. Among 1143 cases, 258 were infected only with HBoV, of which 249 (21.8%) had RTI and 9 were asymptomatic. One patient was diagnosed with plastic bronchitis associated with HBoV and his case was described separately (Section 3.5). All 249 of patients underwent an additional microbiological and diagnostic assessment based on a clinical judgement. After diagnostic work-up, a total of 30 patients had additional conditions or infections (Figure 1). When reporting white blood cell (WBC) and C-reactive protein (CRP) levels, these were excluded, as underlying conditions might have influenced these values.

During 2022–2025, 59 (5.2%) patients who had RTI and HBoV detected in respiratory samples were hospitalized, of which 11 were hospitalizations attributable to HBoV alone. The rest had RTI related to HBoV and viral co-infection: 26 with rhinovirus (RV), 15 with respiratory syncytial virus (RSV), eight with adenovirus (AdV), six with human metapneumovirus (hMPV), five with enterovirus (EV), five with SARS-CoV-2, two with, influenza A virus (IAV), and two with parechovirus. Those with viral co-infections were excluded.

### 2.2. HBoV and Viral Co-Infection Detection

HBoV was detected from respiratory specimens, including upper and lower respiratory tract. Upper respiratory tract specimens included nasopharyngeal swabs or aspirates, and throat swabs, while lower respiratory tract specimens included tracheal aspirates and bronchoalveolar lavage fluid samples.

Viral nucleic acids were extracted from 200 µL of each specimen using the *GeneRotex 96 Nucleic Acid Extractor* (Tialong, Xi’an, China) according to manufacturer’s instructions. Detection of viral nucleic acid was carried out by Reverse Transcription Quantitative Polymerase Chain Reaction (RT-qPCR) using a commercial, multiplex PCR kit for respiratory viruses (*Respiratory Viruses 16-well*, AusDiagnostics, Mascot, Australia). The multiplex PCR panel detects the following respiratory viruses: HBoV, AdV, seasonal coronaviruses (HcoV), SARS-CoV-2, human parainfluenza virus (HPIV) types 1–4, IAV, influenza B virus (IBV), RSV-A and RSV-B, HMPV, RV, human parechovirus and EV. *High Plex* platform (AusDiagnostics, Mascot, Australia) suitable for multiplex-tandem PCR (MT-PCR) was used. The platform is based on a two-step nucleic acid amplification. The first step is based on reverse transcription and pre-amplification throughout 18 cycles, followed by the quantitative real-time second amplification step [16].

### 2.3. Clinical, Radiological and Laboratory Data

We report clinical, radiological and laboratory data for patients treated at UHID with HBoV monoinfection to avoid the possible impact of viral and other coinfections on disease severity. Clinical data included the presence of fever, tachypnea, dyspnea, cough, vomiting and/or diarrhea and hypoxia (defined as oxygen saturation levels below 95%). Diagnosis of pneumonia was based on clinical grounds and CXR findings, if performed. Among patients who underwent CXR, radiographic findings were categorized as alveolar consolidation, interstitial infiltrates, atelectasis, or pleural effusion. Laboratory features included WBC count expressed as ×10^9^/L and CRP values expressed as mg/L.

### 2.4. Statistical Analysis

For categorical variables relative and absolute frequencies were reported, while for continuous variables mean, medians, and interquartile ranges were calculated. For comparison of categorical variables Chi-square or Fisher exact test were used as appropriate, while for comparing continuous variables Mann-Whitney U test was used. Normality of distribution for continuous variables was assessed by Shapiro-Wilk test (*p* < 0.05). Comparisons were made regarding age, gender, co-infection rates between late pandemic and early postpandemic period. Additionally, we examined differences in clinical, laboratory and radiological features between patients up to 2 years of age and older. To assess factors related to pneumonia a multivariable logistic regression model was employed. All statistical tests were two-tailed with the significance level set to 0.05. Data were analyzed using R version 4.5.1 (R Development Core Team, Vienna, Austria).

### 2.5. Ethical Approval

The study was approved by the UHID Ethical Committee (Approval no. 01-1349-5-2026). No identifiable patient data were disclosed.

## 3. Results

### 3.1. Demographic Characteristics of Detected Cases

A total of 1143 HBoV cases were detected from respiratory samples at CIPH during a four-year period, of which 377 (33.0%) were detected during the late pandemic period (Table 1). Male cases comprised 55.6% of overall cases, with no differences in gender distribution between the late pandemic and the early postpandemic period (*p* = 0.360). The average age was 24.9 months. The highest proportion of cases was noted in children aged 13 to 24 months old (55.6%), followed by children aged 2 to 5 years (32.5%), and infants aged 7 to 12 months old (7.0%) We did not find differences between the late pandemic and early postpandemic period in terms of age (*p* = 0.718).

### 3.2. HBoV and Viral Co-Detection Rates

During 2022–2025, a total of 4893, 8084, 7278, and 7106 respiratory specimens among pediatric patients were collected at UHID and analyzed at CIPH, with 234 (4.8%), 313 (3.9%), 259 (3.6%), and 337 (4.7%) being HBoV positive, respectively. However, only 23.9% (56/234), 17.3%, 29.0%, and 21.7% of these were HBoV monoinfections. Monthly prevalence of HBoV cases is presented in Figure 2. The highest absolute number of HBoV cases was detected in 2025, although the highest positivity rate was detected in 2022. Highest monthly case count was observed in December 2023, which was the first season following the end of COVID-19 pandemic. Although case numbers decreased during summer months, viral detection persisted throughout the year, demonstrating a marked seasonal distribution, with the highest number of cases observed during winter months, spanning from November to February (Figure 2). Interestingly, during season 2024/2025 peak prevalence occurred in February, compared to previous seasons (2023/2024 and 2022/2023) which peaked in December.

Distribution of viral co-detections associated with HBoV is presented in Table 1. Overall, RV (353/885; 39.9%) was the most frequently co-detected respiratory virus throughout all study seasons, with the highest number of co-detections recorded in 2025 (Figure 3). Following RV, AdV (250; 28.2%), RSV (132; 14.2%) and EV (136; 15.4%) were also frequently detected. RSV, IV, and HMPV co-detections demonstrated significant seasonality, predominantly occurring during winter months, while RV, HPIV and AdV co-detections were observed without a clear seasonal pattern. SARS-CoV-2 co-detections gradually declined following the 2022/23 season.

Co-detections were observed at similar rates in the late pandemic and early post-pandemic period (79.3% vs. 76.5%, *p* = 0.536). Moreover, viral co-detection rates were comparable between both genders, occurring in 393 (77.5%) females and 492 (77.4%) males (*p* = 0.950). Differences were noted in the prevalence of certain viruses between aforementioned periods (Table 1). We found a significant decrease in AdV coinfections (36.8% vs. 23.9%, *p* < 0.001), while simultaneous increase in HPIV3 (3.3% vs. 7.8%, *p* = 0.013), RV (30.4% vs. 44.7%, *p* < 0.001), and IBV (0.7% vs. 3.2%, *p* = 0.019) coinfection rates.

### 3.3. Clinical, Laboratory and Radiological Features Associated with HBoV Infection

We examined data on 249 patients in whom HBoV was the only detected respiratory pathogen (Table 2). Most of the children were healthy; only 10 had pre-existing chronic conditions (4.0%). Common cold was the most prevalent clinical syndrome (148; 56.2%), followed by AOM which occurred in around one-third of children. Pneumonia was diagnosed in 59 patients (23.7%).

Most common symptoms included fever (216; 95.6%) and cough (184; 81.4%). More than one quarter of patients exhibited gastrointestinal symptoms. Of note, these symptoms were more frequent among individuals aged less than 24 months (34.8% vs. 16.0%, *p* = 0.001).

Severe disease requiring oxygen support or admission to Pediatric Intensive Care Unit (PICU) was rarely observed (seven and four cases, respectively). None of those younger than 2 years were admitted to PICU.

### 3.4. Features Associated with Pneumonia

Among pneumonia cases, 29 out of 59 (49.2%) exhibited solely alveolar consolidation. Interstitial infiltrates were the most common radiological feature (28.6%), and only a minority of patients exhibited combined findings (12.4%). 62.7% of pneumonia cases were male compared to 54.2% of non-pneumonia cases (*p* = 0.250).

Diagnosis of pneumonia was significantly more common among those older than 2 years (33.0% vs. 18.1%, *p* = 0.007), as well as alveolar consolidation (41.7% vs. 15.8%, *p* = 0.003). This group also had higher CRP levels (43.4 mg/L vs. 32.6 mg/L, *p* = 0.038). Interstitial infiltrates were more common among those younger than 2 years, although with no significance (33.3% vs. 22.9%, *p* = 0.239). A total of 43 (72.9%) of pneumonia cases received antibiotic treatment: of these 25 had alveolar, 10 had interstitial and eight had combined radiologic features.

To assess factors associated with clinical diagnosis of pneumonia among HBoV monoinfected patients we employed logistic regression. Among those with pneumonia included in the model, five had coinfections. Variables were used based on clinical significance and internal data analysis. In multivariable analysis, age older than 24 months old (aOR: 2.32; 95% CI: 1.21–4.52; *p* = 0.012) and higher WBC (aOR: 1.13; 95% CI: 1.07–1.20; *p* < 0.001) were significant factors associated with pneumonia (Table 3).

### 3.5. A Case of HBoV-Associated Plastic Bronchitis

Plastic bronchitis is a rare clinical syndrome characterized by the formation of bronchial casts that obstruct the airways, impair ventilation, and may lead to acute respiratory failure. It is more commonly reported among male children. Its pathophysiology remains incompletely understood, and the literature is largely limited to individual case reports and small case series, making its true prevalence difficult to determine [17]. HBoV1 infection has also been described in children with plastic bronchitis in the literature, generally with favorable outcomes [7].

A previously healthy 4-year-old boy presented to the ED on the first day of illness with high-grade fever (up to 40.2 °C) and cough. On admission, his oxygen saturation was 90–94% on room air, heart rate was 164 beats/min, respiratory rate was 40 breaths/min, and tympanic temperature was 39.1 °C. Physical examination revealed diminished breath sounds over the left lung and mildly prolonged expiration. Owing to hypoxemia and tachydyspnea, he was admitted for further diagnostic evaluation and treatment.

Initial laboratory investigations demonstrated WBC of 10.0 × 10^9^/L with neutrophil predominance (85.9%), CRP of 14.6 mg/L, procalcitonin of 0.538 ng/mL, while the remaining biochemical parameters were within normal limits. Chest radiography showed a suspected left retrocardiac basal pulmonary infiltrate with possible minimal pleural reaction.

The patient was initially treated with intravenous ceftriaxone, supplemental oxygen, inhaled racemic epinephrine, salbutamol, corticosteroids, and upper airway toilet. Despite these measures, his respiratory status deteriorated, with progressive hypoxemia and tachydyspnea, necessitating transfer to the PICU on the second day of hospitalization. Repeat chest radiography demonstrated complete homogeneous opacification of the left hemithorax with ipsilateral mediastinal shift, consistent primarily with left lung atelectasis, and a small pleural effusion on the left side (Figure 4A).

Flexible bronchoscopy performed the same day revealed complete obstruction of the left bronchial tree. Cytological analysis of bronchoalveolar lavage revealed a total cell count of 1440 cells/mm^3^ and an erythrocyte count of 250 cells/mm^3^. The patient was subsequently transferred to a collaborating tertiary center, where rigid bronchoscopy was performed and a cohesive bronchial cast forming a complete mold of the left tracheobronchial tree was successfully extracted, confirming the diagnosis of *bronchitis plastica* (Figure 4B). Follow-up chest radiography demonstrated partial re-expansion of the left lung with persistent inflammatory changes involving the left middle and lower lung fields (Figure 4C). Following bronchial cast extraction, supportive treatment was continued, and the patient fully recovered and was discharged home on the seventh day of hospitalization. Extensive microbiological investigations were negative, except for HBoV detected by PCR from nasopharyngeal swab.

## 4. Discussion

The aim of this study was to analyze the epidemiological trends and clinical spectrum of HBoV infection among pediatric patients over a four-year period. Overall, we observed a low and relatively stable HBoV prevalence, ranging from 3.6% to 4.8%. This is lower and less variable than rates reported from other European countries, where HBoV prevalence estimates range from 2.0% in Finland to 21% in Denmark, with the pooled prevalence estimated at 9.57% [1,2]. Studies addressing HBoV epidemiology in the Croatian pediatric population remain relatively scarce and restricted to hospitalized patients [10,11,14,15]. Although previous studies were also conducted in the pediatric population from northwestern Croatia, our study included children receiving both inpatient and outpatient care. Compared to studies from 2016 and 2021, where HBoV prevalence was 10.5% and 7.6%, significantly higher prevalence was found in 2019 study, which included only children <3 years-old diagnosed with LRTI [10,14,15]. The majority of patients were younger than 2 years old, which is in line with previous observations that the burden of HBoV is the greatest in that age group [5,18,19]. The lower HBoV detection rates observed in our study might, at least in part, be explained by disruptions in respiratory virus circulation associated with the COVID-19 pandemic and highlight the importance of ongoing surveillance.

Seasonal patterns of HBoV circulation may differ across climatic regions, as in temperate climate increased HBoV activity has predominantly been reported during the winter and spring months, while in subtropical regions bimodal peaks during summer and winter have been described [20,21]. A similar seasonal pattern was observed, with the highest HBoV activity recorded during the winter months (Figure 2). Our data suggest a potential gradual shift in seasonality, as the 2024/2025 epidemic peaked in February, and similar might be expected in the following season (2025/2026) as case counts observed in December 2025 were lower compared to the same month in previous years. However, this possible shift will be clarified through ongoing surveillance in the following seasons.

Although there is compelling evidence that HBoV is a true respiratory pathogen, several challenges persist in determining causality between HBoV detection and clinical manifestations of RTI. Frequent coinfections with other respiratory pathogens, prolonged viral shedding, and incompletely understood duration of protective immunity still represent the main unresolved issues regarding its pathogenicity [2,12,22,23,24]. However, we still believe that the role of HBoV as a relevant pathogen in RTIs should not be underestimated, given a substantial number of patients infected solely with HBoV in our cohort presenting with common features of both URTI and LRTI.

In our cohort co-detection of one or more respiratory viruses ranged from 71.0% to 82.7%. Co-detection rates varied between age groups, with the highest rates emerging among children aged one to five years old (80% among 13–24 months old, and 78.2% among 2–5 years old), who also accounted for the highest number of HBoV cases. Due to small case counts among those younger than 6 months old, observations regarding co-detections in this age group are inconclusive, and probably reflect secondary household contacts from family members. The estimated respiratory viral co-infection rate among HBoV cases in Europe is 52.4%, with comparably high rates across diverse geographical regions suggesting that viral co-infection is a common feature of HBoV infection [1,2,25,26,27]. RV, AdV, and RSV are the most common pathogens co-detected worldwide [2,28,29]. In Croatia, co-detection rates seem to be preserved in the ongoing period, with RV and AdV still being most frequently detected pathogens [10]. This is one of the first reports assessing longitudinal changes in HBoV co-detection rates among pediatric patients. While a study from Cetin et al. reported a decrease in HBoV co-detections during 2022–2024, we did not document longitudinal differences in overall co-detection rates [30]. Interestingly, we found a decrease in HBoV-AdV and an increase in HBoV-RV co-detection during early post-pandemic compared to late pandemic period (Table 1). Despite being non-enveloped viruses similar to HBoV, both AdV and RV exhibit year-round activity [31]. The significantly higher rate of HBoV–AdV co-detection during the late pandemic period may partly reflect the resurgence of AdV following the relaxation of pandemic-related non-pharmaceutical interventions, followed by a gradual stabilization of virus circulation patterns [32]. This interpretation remains speculative, as the seasonality of AdV circulation remains incompletely defined and uncertain compared to seasonal respiratory viruses such as RSV and influenza [33]. A rise in HBoV-RV co-detections is not fully consistent with the available literature, according to which RV appears to be relatively resilient to the disruptions caused by the COVID-19 pandemic, with an early resurgence and relatively stable circulation patterns [34,35].

In our cohort, HBoV was repeatedly positive in 61 cases. Among these, 26 patients had repeated HBoV detection at least 14 days apart from the primary event and during the same season, with the longest presumed shedding interval of 291 days which was observed in an immunocompetent, 10-months old male. Nevertheless, our findings should be cautiously interpreted, as testing was not performed according to a predefined sampling protocol, but was rather driven by clinician’s assessment during subsequent ED visits. Additionally, most of these patients had documented viral co-detections and non-specific respiratory symptoms, which precludes a reliable attribution of subsequent respiratory episodes to HBoV. Prolonged detection of HBoV DNA in respiratory specimens has previously been described in studies employing different study designs and sampling strategies, with the longest duration of shedding reaching 402 days [12,23,36]. Conventional and quantitative PCR are highly sensitive and widely used, but the prolonged detection of HBoV DNA limits their ability to distinguish acute infection from prolonged viral persistence. A recent prospective study from Slovenia further highlighted this diagnostic challenge by assessing HBoV1 mRNA as a marker of active viral replication. Among children hospitalized with acute bronchiolitis who had HBoV1 DNA detected in nasopharyngeal samples, only a subset was also positive for HBoV1 mRNA, supporting the potential value of mRNA detection in distinguishing active infection from prolonged DNA persistence [37]. As the duration of protective immunity following HBoV-1 infection also remains incompletely understood, distinguishing prolonged shedding from reinfection based solely on repeated PCR positivity is uncertain [22]. These findings suggest that a more accurate assessment of acute HBoV infection may require a multimodal diagnostic approach combining quantitative viral load with markers of active replication and, where appropriate, serological testing.

We observed significant discrepancy between HBoV positive patients and severe disease attributable to HBoV. Among individuals infected solely with HBoV, only 11 (4.4%) were hospitalized, seven (2.8%) required oxygen, and four (1.6%) were admitted to PICU. On the contrary, one of the largest single center studies comprising 1215 HBoV monoinfection cases found a much higher rate of severe illness, with hospital and PICU admission rates being 25.9% and 8.1%, respectively [38]. However, higher admission rates might be partly explained by a great proportion of children with significant comorbidities (25.4%) compared with our cohort involving 4.0% of children with pre-existing comorbidities.

The role of HBoV in acute gastroenteritis has been well established, with estimated prevalence of HBoV positive stool samples ranging from 1.3 to 58.3% worldwide [2]. Gastrointestinal manifestations were also relatively common in our cohort. Despite the relatively high rate of gastrointestinal symptoms observed in our cohort, causal association with HBoV remains unclear, as stool samples were not systematically analyzed, gastrointestinal manifestations were not equally prominent across patients, and stool samples were not tested for HBoV. Among 58 cases with gastrointestinal symptoms, in eleven patients stool analysis was performed, including immunochromatographic testing for rotavirus and adenovirus and stool culture, which came negative.

In our cohort almost one-third of patients had otoscopy findings consistent with AOM. Contrarily, Nokso-Koivisto et al. have reported 52% AOM cases among HBoV infected children [39]. According to Beder et al., among 222 AOM cases, HBoV-1 was detected in 6.3% of samples suggesting a potential role of HBoV1 in facilitating secondary pneumococcal infection [40]. However, the underlying mechanisms remain incompletely understood and warrant further investigation. We found a relatively frequent antibiotic use (33.7%), which is consistent with previous studies and likely reflects clinical uncertainty regarding concomitant bacterial infection and the difficulty of establishing a definitive disease etiology using specimens available in routine practice [3,11]. Even higher antibiotic treatment was noted among pneumonia cases, additionally highlighting this issue. Despite the freuqent use of antibiotics, bacterial pathogens were isolated from upper and lower respiratory tract specimens in only eleven (4.4%) patients: *S. pneumoniae* in six, non-typable *H. influenzae* and *S. aureus* in two, and *M. catarrhalis* in one case. Of those, two patients had invasive pneumococcal disease, with *S. pneumoniae* detected from primary sterile sites (blood and empyema samples). Additionally, one patient had *M. pneumoniae*. According to the current literature, *S. pneumoniae*, *M. catarrhalis*, and *H. influenzae* are most commonly reported bacterial isolates in HBoV patients, although detection methods in these studies vary [3,41]. While bacterial etiology of alveolar pneumonia has been firmly established, evidence of the role of bacterial agents, especially *S. pneumoniae* in non-alveolar pneumonia remains inconclusive, and might at least in part contribute to higher antibiotic use [42].

Several studies so far have addressed the role of HBoV in pneumonia among pediatric patients, either as a sole pathogen or as in co-infection with other respiratory pathogens [19,43,44,45]. We found higher WBC and older age as the only factors associated with pneumonia, while CRP levels were only significant in univariate analysis. Li et al. found no association with age among severe and non-severe HBoV pneumonia cases [43]. Interestingly, among 370 cases of HBoV pneumonia, the highest incidence of severe pneumonia was found among patients older than 2 years [46]. We observed a higher prevalence of interstitial infiltrates among younger children, although with no statistical significance. Nevertheless, differences in radiological features might indicate different disease phenotypes across age groups, which possibly might be related to complex processes of waning transplacental immunity, as well as changing epidemiological circumstances including daycare attendance [26]. So far, studies investigating immunological mechanisms underlying severe pneumonia are scarce and failed to establish firm associations, even across different age groups [42,43]. A high proportion of alveolar infiltrates among pneumonia cases and association with higher leukocyte count suggest that the majority of pneumonia cases ocurred in the setting of bacterial co-infection, while primary viral pneumonia associated with HBoV is rather rare. However, given the small subset of pneumonia cases, these findings should be cautiously interpreted, especially as viral load measurements were not available.

Several limitations should be acknowledged. Routine RT-PCR does not provide information of HBoV type which would provide a more detailed pathogen characterization. Moreover, distinguishing disease attributable to HBoV, especially in cases where HBoV is not the only pathogen remains challenging, particularly due to evidence of prolonged shedding. Obtaining RT-PCR cycle threshold values would help clarify the contribution of HBoV to clinical features.

## 5. Conclusions

This is the largest single-center study to date investigating the epidemiological and clinical characteristics of HBoV infection in the general pediatric population in Croatia. HBoV infection predominantly affects toddlers and is less frequently encountered in infants and school-aged children. Viral co-infections were common and show evolving trends.

Pneumonia appears to be a frequent clinical manifestation of HBoV infection, with higher prevalence observed among older children. High rates of antibiotic prescription highlight diagnostic challenges in distinguishing HBoV-attributable pneumonia from pneumonia arising as a secondary complication, posing challenges in terms of antimicrobial stewardship.

Further studies are needed to better define the independent contribution of HBoV to disease severity and clinical outcomes, particularly in the setting of viral and bacterial co-infections.

## Figures and Tables

**Figure 1:**
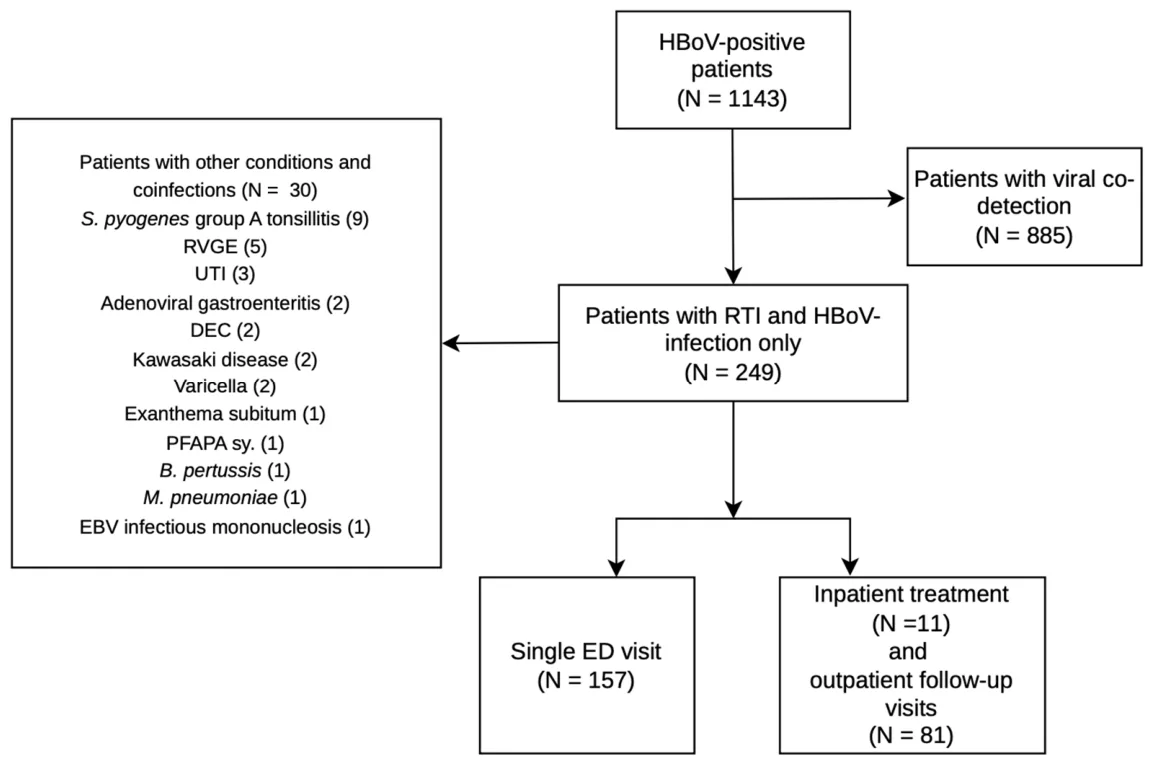
Flowchart of HBoV-positive patients classification according to viral co-infection status, and co-occurring infections and conditions. DEC—Diarrheagenic *E. coli*; EBV—Epstein-Barr virus; ED—Emergency Department; PFAPA—Periodic Fever, Aphthous Stomatitis, Pharyngitis, and Cervical Adenitis; RTI—Respiratory Tract Infection; RVGE—Rotaviral Gastroenteritis; UTI—Urinary Tract Infection.

**Figure 2:**
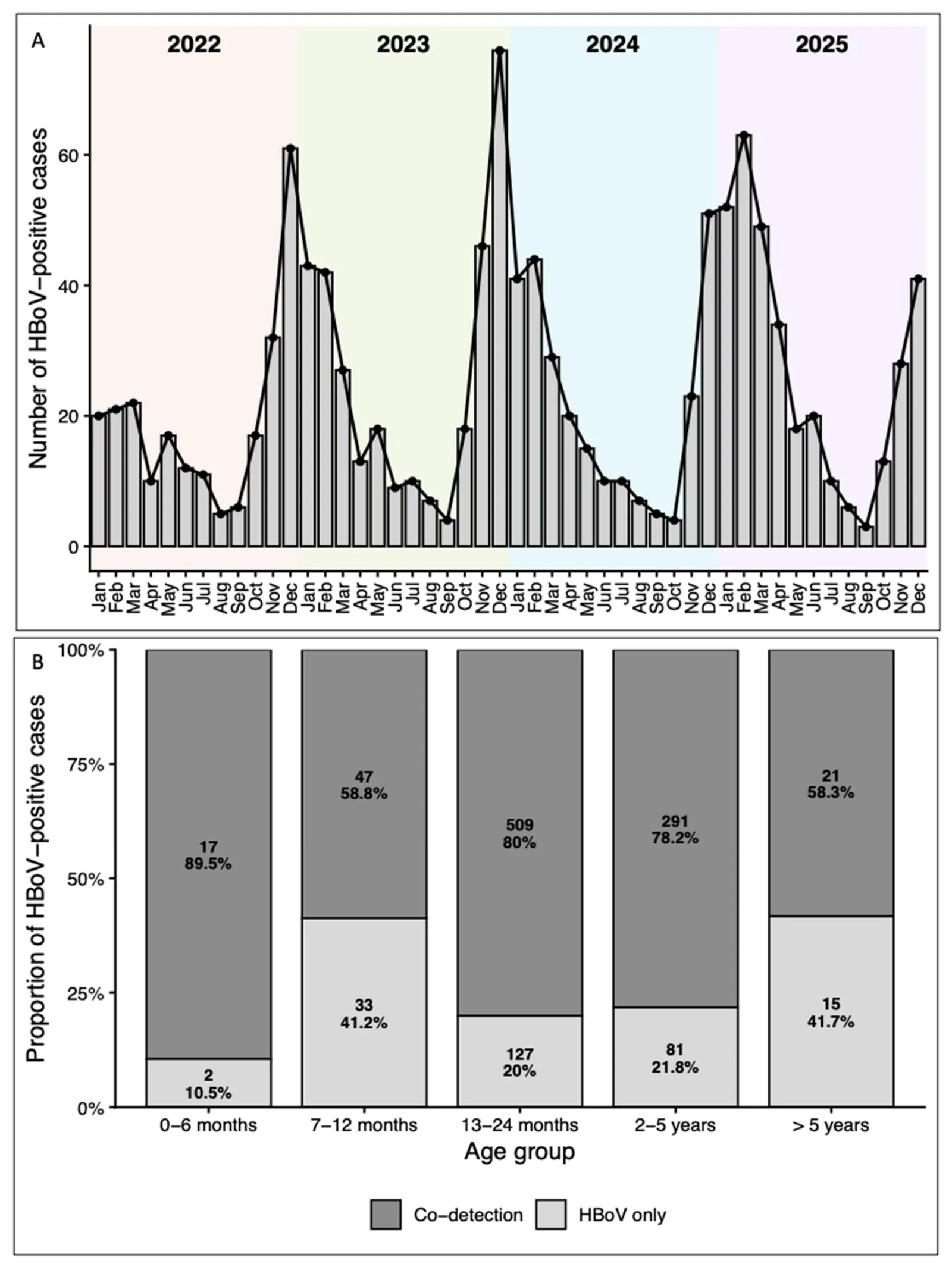
(**A**) Trends of HBoV infection during the studied period; (**B**) Proportions of viral co-detections among different age groups.

**Figure 3:**
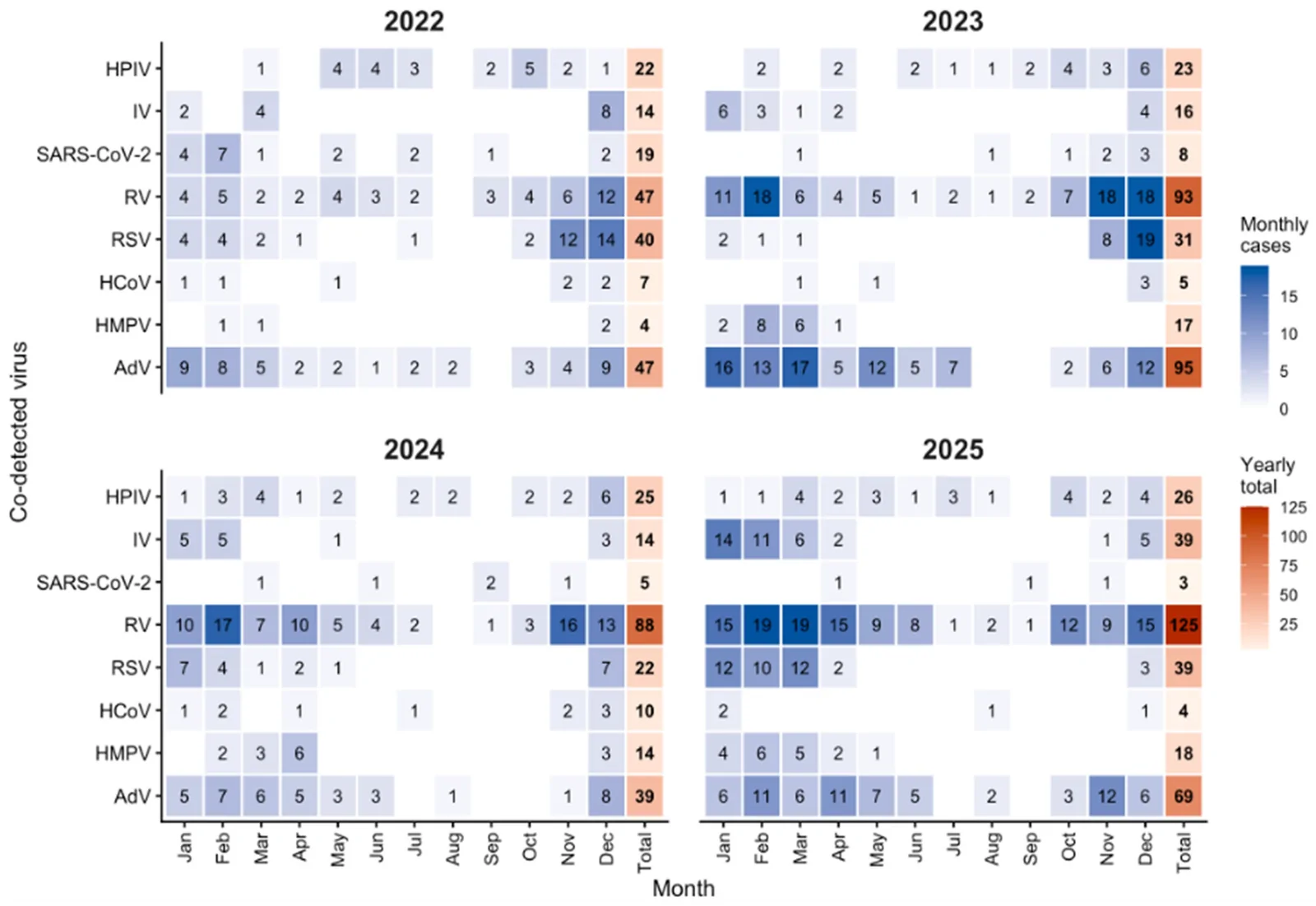
Co-detection rates of respiratory viruses among patients with PCR-proven HBoV infection during the studied period.

**Figure 4:**
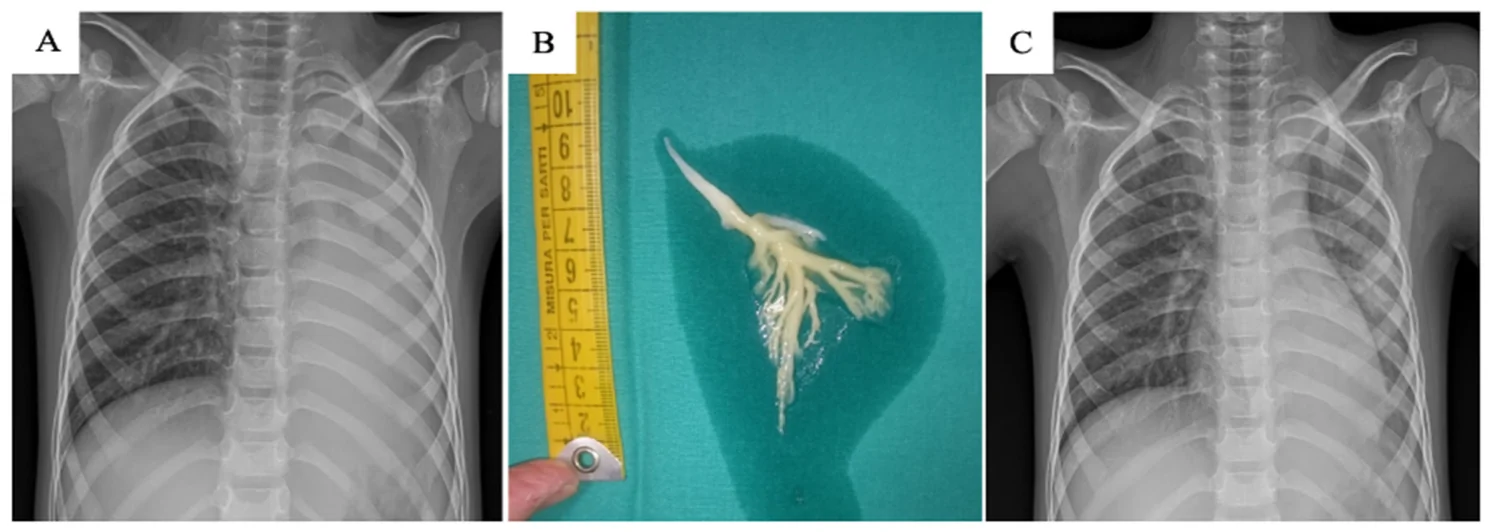
(**A**) Homogeneous opacification of the left hemithorax with ipsilateral mediastinal shift, consistent with extensive left lung atelectasis; (**B**) Bronchial casts retrieved during bronchoscopy; (**C**) Chest X-ray obtained after bronchoscopy demonstrating an immediate resolution of previously observed abnormalities, with only residual retrocardiac left basal opacities.

**Table 1:** Baseline characteristics of HBoV-positive cases.

	2022–2025 N (%)	Late Pandemic Period ** N (%)	Early Post-Pandemic Period N (%)	*p*
**Total**	1143	377 (33.0)	766 (67.0)	-
**Gender**				
Males	636 (55.6)	217 (57.6)	419 (54.7)	0.360
Females	507 (44.4)	160 (42.4)	347 (45.3)	
**Age group**				
≤6 months old	19 (1.7)	6 (1.6)	13 (1.7)	
7–12 months old	80 (7.0)	23 (6.1)	57 (7.4)	0.583
1–2 years old	636 (55.6)	211 (56.0)	425 (55.5)	
2–5 years old	372 (32.5)	121 (32.1)	251 (32.8)	
>5 years old	36 (3.2)	16 (4.2)	20 (2.6)	
**Age (months)**				
Mean	24.9	25.4	24.8	0.718
Median (IQR)	20.5 (15.8–29.3)	20.3 (15.9–30.6)	20.6 (15.7–29.0)	
**HBoV only**	258 (22.6)	78 (20.7)	180 (23.5)	0.286
**Any viral co-detection**	885 (77.4)	299 (79.3)	586 (76.5)	
**Co-detected viruses**				
One virus	615 (53.8)	203 (53.8)	412 (53.8)	0.599
Two viruses	246 (21.5)	89 (23.6)	157 (20.5)	
≥three viruses	24 (2.1)	7 (1.9)	17 (2.2)	
AdV	250 (28.2 ^†^)	110 (36.8)	140 (23.9)	<0.001
EV	136 (15.4)	47 (15.7)	89 (15.2)	0.836
HMPV	53 (6.0)	21 (7.0)	32 (5.5)	0.354
HPIV 1–4 *	96 (10.8)	26 (8.7)	70 (11.9)	0.142
HPIV 1	14 (1.6)	8 (2.7)	6 (1.0)	0.085
HPIV 2	8 (0.9)	5 (1.7)	3 (0.5)	0.128
HPIV 3	56 (6.3)	10 (3.3)	46 (7.8)	0.009
HPIV 4	19 (2.1)	3 (1.0)	16 (2.7)	0.139
Influenza viruses	83 (9.4)	26 (8.7)	57 (9.7)	0.618
Influenza virus A	62 (7.0)	24 (8.0)	38 (6.5)	0.395
Influenza virus B	21 (2.4)	2 (0.7)	19 (3.2)	0.018
HCoVs	26 (2.9)	9 (3.0)	17 (2.9)	1.000
SARS-CoV-2	35 (4.0)	20 (6.7)	15 (2.6)	0.005
Parechoviruses	17 (1.9)	8 (2.7)	9 (1.5)	0.300
RV	353 (39.9)	91 (30.4)	262 (44.7)	<0.001
RSV	132 (14.9)	44 (14.7)	88 (15.0)	0.905

* one patient had HPIV3 and HPIV4 co-infection; ** from 1 January 2022 to 31 May 2023; ^†^ percentages were calculated among patients with co-detections (n = 885); AdV—adenovirus; EV—enterovirus; HCoVs—Human Coronaviruses; HMPV—Human Metapneumovirus; HPIV—Human parainfluenza virus; RV—Rhinovirus; RSV—Respiratory Syncytial Virus; SARS-CoV-2—Severe Acute Respiratory Syndrome Coronavirus-2.

**Table 2:** Demographic, clinical, laboratory, and radiological features of patients in whom HBoV was the only detected pathogen.

	2022–2025 N (%)	Age Less Than 24 Months N (%)	Older Than 24 Months N (%)	*p*
**Total cases**	249 (100.0)	155 (62.2)	94 (37.8)	-
**Gender**				
Male	140 (56.2)	91 (58.7)	49 (52.1)	0.310
Female	109 (43.8)	64 (41.3)	45 (47.9)	
**Comorbidities**	10 (4.0)	4 (2.6)	6 (6.4)	
Neurologic	1 (10.0)	1 (25.0)	-	
Pulmonary	5 (50.0)	3 (75.0)	2 (33.3)	
Genetic disorders	3 (30.0)	0 (0.0)	3 (50.0)	
Other	1 (10.0)	0 (0.0)	1 (16.7)	
**Clinical syndromes ^†^**				
Common cold	148 (59.4)	98 (63.2)	50 (53.2)	0.118
Croup	13 (5.2)	5 (3.2)	8 (8.5)	0.082
Acute otitis media	76 (30.5)	50 (32.2)	26 (27.7)	0.445
Bronchitis/bronchiolitis	23 (9.2)	17 (11.0)	6 (6.4)	0.226
Pneumonia	59 (23.7)	28 (18.1)	31 (33.0)	0.007
**Leukocyte count (×10^9^/L)**				
Mean	12.6	12.7	12.5	0.852
Median (IQR)	11.7 (8.4–15.3)	11.6 (8.4–15.6)	11.8 (8.5–14.8)	
**CRP value (mg/L)**				
Mean	36.8	32.6	43.4	0.038
Median (IQR)	15.2 (5.1–50.2)	12.3 (4.3–31.7)	19.1 (6.9–68.1)	
Fever	235 (94.4)	144 (92.9)	91 (96.8)	-
Tachypnea	16 (6.4)	9 (5.8)	7 (7.4)	0.609
Dyspnea	20 (8.0)	10 (6.5)	10 (10.6)	0.239
Cough	199 (79.9)	122 (78.7)	77 (81.9)	0.541
Vomit and/or diarrhea	69 (27.7)	54 (34.8)	15 (16.0)	0.001
SpO2 < 95% on admission	18 (7.2)	8 (5.2)	10 (10.6)	0.106
**CXR performed**	105 (42.2)	57 (36.8)	48 (51.1)	0.027
Alveolar consolidation	29 (27.6)	9 (15.8)	20 (41.7)	0.003
Interstitial infiltrates	30 (28.6)	19 (33.3)	11 (22.9)	0.239
Combined infiltrate	13 (12.4)	6 (10.5)	7 (14.6)	0.565
Pleural effusion	9 (8.6)	2 (3.5)	7 (14.6)	0.076
Atelectasis	2 (1.9)	0 (0.0)	2 (4.2)	0.211
**Oxygen requirement**	7 (2.8)	2 (1.3)	5 (5.3)	0.108
**Antibiotic treatment**	84 (33.7)	50 (32.3)	34 (36.2)	0.527
**PICU admission**	4 (1.6)	0 (0.00)	4 (4.3)	0.019

^†^ some patients had overlapping clinical syndromes.

**Table 3:** Factors associated with clinical diagnosis of pneumonia among HBoV-positive individuals.

Variable (*)	OR	*p*	aOR	*p*
Gender (female)	1.42 (0.79–2.62)	0.251	1.54 (0.79–3.06)	0.206
Age (<24 months)	2.23 (1.23–4.06)	0.008	2.32 (1.21–4.52)	0.012
Tachypnea (absent)	4.71 (1.67–13.77)	0.003	1.90 (0.33–12.00)	0.472
Dyspnea (absent)	3.67 (1.43–9.45)	0.006	2.30 (0.41–11.68)	0.314
CRP (per 10 mg/L)	1.07 (1.02–1.13)	0.005	1.02 (0.96–1.08)	0.500
WBC (per 1 × 10^9^/L)	1.12 (1.07–1.19)	<0.001	1.13 (1.07–1.20)	<0.001

* reference category.

## Data Availability

Data is available from the corresponding author upon a reasonable request.

## Abbreviations

AdV	Adenovirus
AOM	Acute Otitis Media
CIPH	Croatian Institute of Public Health
COVID-19	Coronavirus Disease 2019
CRP	C-reactive Protein
IAV	Influenza A Virus
IBV	Influenza B Virus
HBoV	Human Bocavirus
HCoVs	Human Coronaviruses
HPIV	Human Parainfluenza Virus
LRTI	Lower Respiratory Tract Infection
MT-PCR	Multiplex Tandem Polymerase Chain Reaction
RTI	Respiratory Tract Infection
RV	Rhinovirus
RSV	Respiratory Syncytial Virus
RT-qPCR	Reverse Transcription Quantitative Polymerase Chain Reaction
UHID	University Hospital for Infectious Diseases
URTI	Upper Respiratory Tract Infection
WBC	White Blood Cell
